# Inflamed endothelial cells express S1PR1 inhibitor CD69 to induce vascular leak

**DOI:** 10.1016/j.jbc.2025.110455

**Published:** 2025-07-04

**Authors:** Michel V. Levesque, Andreane Cartier, Yueh-Chien Lin, Raj Kumar Sah, Hanming Zhang, Balkhrisa Chaube, Mantu Bhaumik, Jakob Körbelin, Yajaira Suárez, Carlos Fernández-Hernando, Timothy Hla

**Affiliations:** 1 Vascular Biology Program, Boston Children’s Hospital, Department of Surgery, Harvard Medical School, Boston, Massachusetts, USA; 2 Vascular Biology and Therapeutics Program, Yale Center for Molecular and Systems Metabolism, Department of Comparative Medicine, Yale University School of Medicine, New Haven, Connecticut, USA; 3 IDDRC Mouse Gene Manipulation Core, Neurobiology program, Boston Children’s Hospital, Boston, Massachusetts, USA; 4 Department of Oncology and Hematology & Bone Marrow Transplantation, University Medical Center Hamburg-Eppendorf, Hamburg, Germany

**Keywords:** endothelial cell, inflammation, CD69, sphingosine-1-phosphate (S1P), receptor internalization, tumor necrosis factor α (TNFα), permeability, tight junction

## Abstract

Inflammation disrupts endothelial barrier function and causes vascular leak into the tissue parenchyma. Sphingosine 1-phosphate receptor-1 (S1PR1) in endothelial cells (ECs) is a key inducer of endothelial junctions and barrier function. We report here that ECs activation by the cytokine TNFα and TLR3 agonist polyinosine/polycytosine (pI:C) induces the lymphocyte activation molecule CD69 *via* the canonical NFκB pathway. EC CD69 stimulates endocytosis of S1PR1, inhibits its downstream intracellular signaling events and barrier function. Administration of TLR4 or TLR3 agonists or intranasal infection of mouse-adapted influenza virus (H1N1) or coronavirus (MHV-A59) induced CD69 in lung ECs. Adeno-associated virus-mediated overexpression of CD69 in lung EC leads to decreased cell-surface expression of S1PR1 and tight junction protein claudin-5, concomitantly with increased vascular permeability in the lungs. Furthermore, lung vascular leak at the peak of H1N1 infection is attenuated in a genetic mouse model which lacks CD69 in the endothelium. These data suggest that endothelial activation during inflammation and viral host-defense induces CD69 which downregulates S1PR1 to induce vascular leakage. CD69 induction during endothelial dysfunction may drive exaggerated inflammation by antagonizing the endothelial protective S1PR1 pathway.

Aberrant cytokine or pathogen-associated molecular pattern activation of the endothelium occurs during respiratory viral infections and systemic inflammatory syndromes (1, 2). In such situations, normal functions of the endothelium are compromised, leading to organ dysfunction, morbidity, and mortality of the organism. A key endothelial function that is compromised during pathologic endothelial cell (EC) activation is loss of barrier function which leads to vascular leak (3). The ensuing permeability of plasma components into tissue spaces initiates acute inflammatory events needed for pathogen elimination, subsequent resolution, and wound healing mechanisms that return the tissues to homeostasis. EC-intrinsic protective mechanisms restrain excessive vascular leak and therefore promote vascular health.

The sphingosine-1-phosphate (S1P) receptor-1 (S1PR1) expressed abundantly in EC is one such EC protective pathway that maintains the barrier function of most vascular beds (4, 5). EC specific genetic deletion of *S1pr1* (*S1pr1-ECKO*) or the pharmacological inhibition of S1PR1 induces vascular leakage in mice, which drives pulmonary fibrosis, especially in aged mice (6, 7, 8, 9). It has also been proposed that endothelial S1PR1 activation reduces the cytokine amplification and immune cell recruitment into the lung in an influenza mouse model (10). Mechanisms that regulate endothelial S1PR1 during inflammation and host–pathogen interactions are not known.

CD69, a member of the C-type lectin domain protein family, is a cell-surface type II membrane glycoprotein (11). CD69 was first characterized as an early activation marker on lymphocytes (12). A key function of CD69 is to inhibit egress of activated lymphocytes by suppressing S1PR1 function (13). Specifically, CD69 interacts with S1PR1 and promotes S1PR1 internalization, therefore preventing lymphocytes to sense the extracellular S1P gradient which is an egress signal for lymphocyte trafficking (14, 15). Whether CD69 is expressed and functions in the endothelium is not known.

Here, we show that inflammatory cytokines, toll-like receptor (TLR) agonists and respiratory viral infections induce CD69 expression in ECs. We also found that CD69 expression is restrained by the endothelial transcription factor ETS-related gene (ERG). The presence of CD69 at the surface of ECs downregulates S1PR1 and blocks its function which promotes vascular leak. Our results suggest that inflammatory endothelium represses S1PR1-induced vascular barrier functions by inducing the CD69 protein.

## Results

### CD69 induction by TNF**α** in EC

**Figure 1:**
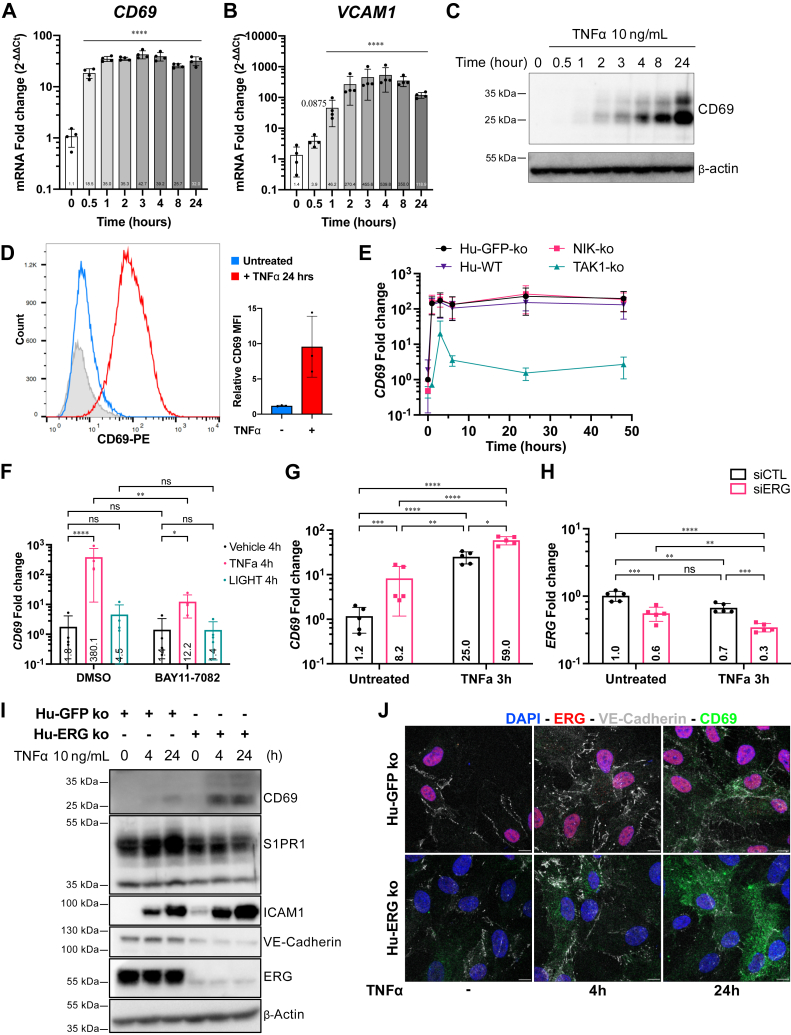
**CD69 expression in ECs *in vitro*, and regulation by NFKB and ERG**. CD69 (*A*) and VCAM1 (*B*) mRNA expression in HUVEC were quantified by reverse transcription-quantitative real-time PCR (RT-qPCR) assay as described. Gene expression is presented as fold change (2-ΔΔCt) of control untreated cells from four independent experiments. Statistical analysis was performed on ΔΔCt values using a one-way ANOVA followed by a Tukey test for each time points to the untreated condition. *C*, immunoblot analysis of TNFα treated HUVEC for CD69 protein expression. A representative blot of at least three independent experiments is presented. *D*, flow cytometry analysis showing CD69 surface expression of HUVEC treated or not with TNFα for 24 h. Unstained cells were used to define background signal (*gray*). The graph shows the normalized CD69 MFI (mean fluorescence intensity of CD69 over unstained control) for four independent experiments. An unpaired *t* test statistical analysis was performed. *E*, RT-qPCR analysis of CD69 mRNA expression in HUVEC CRISPR/Cas9 knockout for NIK (NIK-ko) and TAK1 (TAK1-ko). HUVEC expressing a gRNA targeting GFP (GFP-ko) and parental HUVEC (no transduction) were use as controls. Cells were treated with 10 ng/ml of TNFα for different times. CD69 expression is presented as change (2-ΔΔCt) of control untreated cells and was analyzed with Holm-Šídák’s multiple comparison test. *F*, RT-qPCR analysis of CD69 mRNA expression in HUVEC pretreated for 1 h with 10 μM BAY11 to 7082 or DMSO (diluent) followed by treatment with vehicle (*black*), 10 ng/ml TNFα (*pink*) or 100 ng/ml LIGHT (*green*) for 4 h. CD69 fold change was analyzed with two-way ANOVA followed by Holm-Šídák’s multiple comparison test. RT-qPCR analysis of CD69 (*G*) and ERG (*H*) mRNA expression in HUVEC transfected with siRNA control (siCTL) or siRNA targeting ERG (siERG). After 48 h post transfection, cells were treated 3 h with TNFα or vehicle (untreated) prior to total RNA harvest. Gene expression is presented as fold change (2-ΔΔCt) of control untreated cells. Statistical analysis from five independent experiments was performed on ΔΔCt values using a two-way ANOVA followed by a *post hoc* Holm-Šídák’s multiple comparison test. *I*, immunoblot analysis of HUVEC CRISPR/Cas9 knockout for ERG (ERG-ko) and GFP (control, GFP-ko) treated with TNFα, 10 ng/ml for the specified times. The immunoblots for CD69, S1PR1, ICAM1, VE-cadherin, ERG, and β-Actin are presented. Representative images of three independent experiments are shown. *J*, confocal microscopy of HUVEC CRISPR/Cas9 knockout for ERG (ERG-ko) and GFP (control, GFP-ko) treated with TNFα, 10 ng/ml for the specified times. Cells were stained for CD69 (*green*), ERG (*red*), VE-cadherin (*white*), and DAPI (*blue*). (The scale bar represents 10 μm). Representative images of five separate areas taken randomly are shown. ns = not significant; ∗ = *p* < 0.05; ∗∗ = *p* < 0.01; ∗∗∗ = *p* < 0.001; ∗∗∗∗ = *p* < 0.0001. DAPI, 4',6-diamidino-2-phenylindole; EC, endothelial cell; ERG, ETS-related gene; gRNA, guide RNA; HUVEC, human umbilical vein endothelial cell; RT-qPCR, reverse transcription-quantitative real-time PCR; S1PR1, sphingosine 1-phosphate receptor-1; TNFα, tumor necrosis factor α.

Given the role of CD69 in suppressing S1PR1 function, we reviewed previous EC gene profiling studies and found evidence of *CD69* mRNA expression (16, 17, 18). To confirm these studies, human umbilical vein endothelial cells (HUVEC) were treated with tumor necrosis factor α (TNFα) and *CD69* mRNA was quantified. We observed rapid and sustained induction of *CD69* mRNA by TNFα (Fig. 1*A*) with a similar kinetics of induction as *VCAM1* mRNA (Fig. 1*B*). Immunoblot analysis confirmed the expression of the CD69 polypeptide with similar kinetics as the mRNA (Fig. 1*C*). Flow cytometry experiments on HUVECs stimulated with TNFα showed that TNFα-induced CD69 is expressed on the cell surface (Fig. 1*D*). TNFα also induced CD69 expression in human lung microvascular ECs (Fig. S1*A*). These results confirmed the inflammatory cytokine activation of ECs *CD69* gene expression and accumulation of CD69 protein at the cell surface.

### CD69 expression is induced by NF-**κ**B pathway and repressed by ERG

Since TNFα induces NFκB pathway to regulate gene expression, we checked if canonical (TAK1-dependent) or the noncanonical (NIK-dependent) NFκB pathways are involved (19, 20, 21). Our data show that *CD69* induction was reduced in *TAK1-ko* but remained unchanged in *the NIK-ko* cells (Fig. 1*E*). Similarly, VCAM1 induction was also greatly reduced in *TAK1*-ko HUVEC (Fig. S1*B*). The LIGHT cytokine, which activates the lymphotoxin-β receptor (LTβR) and the downstream noncanonical NF-κB pathway (22), did not induce the *CD69* mRNA levels. Further, BAY 11 to 7082, an IκB kinase (IKK) inhibitor suppressed TNFα induction of *CD69* mRNA (Fig. 1*F*). Comparable results were obtained when analyzing *VCAM1* mRNA expression (Fig. S1*C*). These results suggested that the activation of the canonical NF-κB pathway is critical to induce *CD69* expression in EC *in vitro*.

ERG, a key transcriptional factor important for endothelial lineage gene expression (23, 24, 25), was shown to inhibit the NF-κB pathway (26, 27). We transfected HUVECs with siRNA targeting *ERG* (siERG) or with siRNA control (siCTL). Treatment of siCTL HUVEC with TNFα reduced *ERG* mRNA to about 70% of its initial level. When siERG cells were treated with TNFα, the level of ERG was at its lowest, with about 30% of the control condition. Cells with low *ERG* mRNA levels exhibited higher *CD69* mRNA levels when compared to siCTL cells (Fig. 1, *G* and *H*). After TNFα treatment, the siERG cells showed significant increased expression of CD69 compared to control cells. On the other hand, TNFα-induced *VCAM1*, *ICAM1*, or *SELE* expression was not affected by ERG silencing (Fig. S1, *D*–*F*). Transfection with siERG was sufficient to increase baseline expression of *ICAM1* and *SELE* mRNAs levels. However, no significant differences were observed after TNFα stimulation in both siERG or siCTL conditions. When *ERG* gene was knocked out in HUVEC using a CRISPR/Cas9-mediated gene disruption, TNFα -induction of CD69 was much higher than in the ERG^+^-HUVEC control guide RNA counterparts (Fig. 1, *I* and *J*). Together, our data indicate that the activation NF-κB pathway by TNFα induces CD69 expression in HUVEC and that ERG restrains cytokine induction of *CD69* mRNA.

### CD69 suppresses S1PR1 signaling in ECs

**Figure 2:**
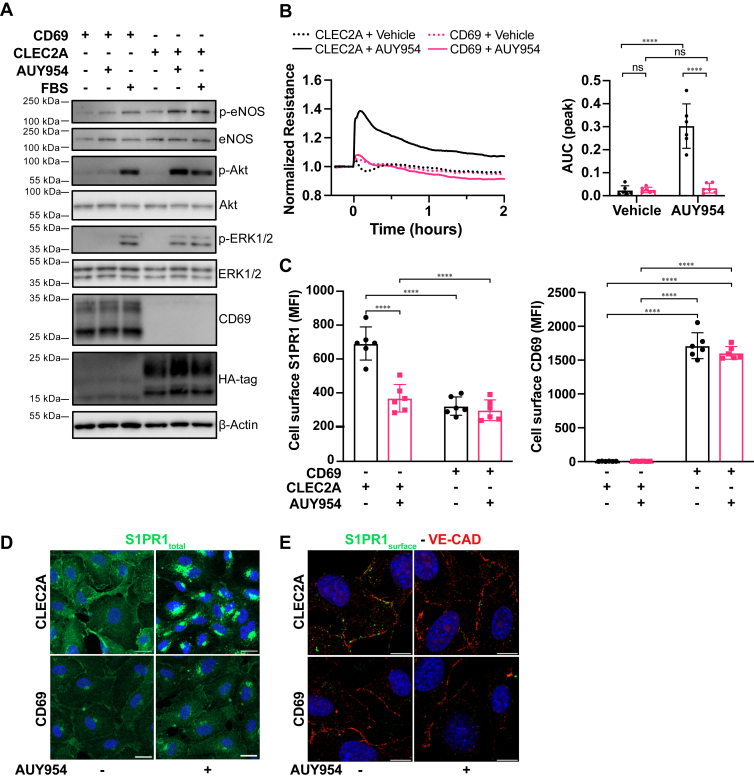
**CD69 expression inhibits S1PR1 signaling and function in HUVEC**. *A*, immunoblot analysis of S1PR1 induced intracellular signaling proteins. HUVECs overexpressing CD69 or CLEC2A were serum starved for 2 h and then stimulated with AUY954 (1 μM) for 5 min. The immunoblots for eNOS, AKT, and ERK1/2 and their phosphorylated forms are presented. Expression of transgenes is detected with CD69 specific and HA-tag antibodies. Representative images of at least three independent experiments are presented. *B*, HUVECs overexpressing CD69 or CLEC2A were analyzed for barrier function by real-time measurement of TEER. At time 0, AUY954 (100 nM) or vehicle (DMSO) was added. For each well, the data were normalized to the resistance prior to stimulation (*graph on the left*). The AUC was calculated for each stimulation peak (*histogram on the right*). Statistical analysis of AUC data of six independent experiments was performed using a two-way ANOVA followed by a *post hoc* Holm-Sidak test for multiple comparisons. *C*, flow cytometry analysis of cell surface expression for S1PR1 and CD69. HUVECs overexpressing CD69 or CLEC2A were stimulated for 1 h with either AUY954 (0.1 μM) or vehicle. Statistical analysis of MFI data from six independent experiments was performed using a two-way ANOVA followed by a *post hoc* Holm-Sidak test for multiple comparisons. *D*, confocal microscopy of total S1PR1 (*green*) in permeabilized HUVECs expressing CD69 or CLEC2A after 1 h of stimulation with AUY954 (1 μM) or vehicle. Cells were fixed and permeabilized prior to staining for S1PR1 and nuclear labeling with DAPI (blue) (the scale bar represents 20 μm). Representative image from four independent experiments is shown. *E*, confocal microscopy of surface S1PR1 (*green*) and total VE-cadherin (*red*) in nonpermeabilized HUVECs expressing CD69 or CLEC2A after 1 h of stimulation with AUY954 (1 μM) or vehicle. Cells were first stained with an antibody recognizing the extracellular domain of S1PR1 using the same conditions used for flow cytometer. The cells were then fixed and permeabilized prior to staining for VE-cadherin and nuclear labeling with DAPI (*blue*) (The scale bar represents 10 μm). Representative image from six separate experiments is shown. ns = not significant; ∗∗∗∗ = *p* < 0.0001. AUC, area under the curve; DAPI, 4',6-diamidino-2-phenylindole; DMSO, dimethyl sulfoxide; HUVEC, human umbilical vein endothelial cell; MFI, mean fluorescence intensity; S1PR1, sphingosine 1-phosphate receptor-1; TEER, transendothelial electrical resistance.

To determine if CD69 expression by ECs regulates S1PR1, we generated HUVEC stably overexpressing CD69 (HUVEC + CD69) using a lentiviral expression system. As control, we used cells overexpressing CLEC2A (HUVEC + CLEC2A) which is in the same protein family as CD69 but lacks the ability to suppress S1PR1 (28). Activation of S1PR1 using a selective agonist, AUY954, induced ERK1/2, AKT, and eNOS phosphorylation in the control cells but not in HUVEC expressing CD69 (Fig. 2*A*). However, fetal bovine serum (FBS)-mediated activation of ERK1/2, AKT, and eNOS was unaltered in both cell populations. These results demonstrate that the S1PR1 intracellular signaling mechanisms are inhibited specifically by CD69.

Since S1PR1 enhances the barrier function in ECs, we measured transendothelial electrical resistance after S1PR1 activation by AUY954 in CD69 and CLEC2A (control) transduced HUVEC. Our data show that S1PR1 activation enhanced transendothelial electrical signal (barrier function) in CLEC2A^+^ cells but not in CD69^+^ HUVEC (Fig. 2*B*). The basal barrier function was similar between CD69 and CLEC2A transduced cells (Fig. S1*G*). Therefore, CD69 expression inhibited the S1PR1 promotion of the barrier function.

In lymphocytes, CD69 has been demonstrated to promote S1PR1 internalization which ultimately leads to its degradation (9, 13, 14, 29). We measured cell surface expression of S1PR1 using flow cytometry. In control cells, S1PR1 surface expression was readily detected (Fig. 2*C*). After treatment of control cells with AUY954 for 1 h, the surface level of S1PR1 was significantly reduced, consistent with its ability to induce endocytosis of S1PR1 (9). HUVECs overexpressing CD69 had significantly reduced surface expression of S1PR1. The remaining signal was unchanged by stimulation with AUY954 treatment. This suggests that CD69 reduced the S1PR1 cell-surface expression to background levels.

Next, we detected S1PR1 on fixed and permeabilized cells by immunofluorescence confocal microscopy (Fig. 2*D*). The level of S1PR1 in permeabilized cells was reduced in CD69 expressing cells compared to the CLEC2A control counterparts. After stimulation with AUY954, S1PR1 signal was associated with perinuclear vesicles in CLEC2A expressing HUVECs. However, in CD69 expressing cells, a weak S1PR1 signal was detected and was minimally affected by the S1PR1 AUY954 treatment. In nonpermeabilized HUVEC, we observed S1PR1^+^ punctate structures localized at or near the cell-cell junctions of HUVEC (VE-cadherin^+^). Junctional S1PR1^+^ punctate structures were markedly reduced by AUY954 treatment. Junctional S1PR1^+^ punctate structures were absent in CD69 expressing cells, with or without AUY954 treatment (Fig. 2*E*). Together these data suggest that CD69 reduces S1PR1 endothelial surface localization near cell-cell junctions, thus suppressing intracellular signaling and vascular barrier function.

The reduced immunofluorescence signal of S1PR1^total^ in the CD69 expressing cells suggests that S1PR1 is internalized and degraded in these cells. To verify that hypothesis, we measured S1PR1 levels in these cells by immunoblot analysis which showed CD69-dependent reduction of S1PR1 protein levels in HUVEC (Fig. S1*H*). In addition to a diminished level of full-length S1PR1 (∼40 kDa), CD69 expressing cells showed a 30 kDa band, which may represent a degradation product of S1PR1. Treatment of these cells with a proteasome inhibitor, MG132, partially rescued S1PR1 suppression (Fig. S1*H*). These data show that CD69 promotes S1PR1 internalization and degradation in EC, which results in reduced signaling capacity of the G protein-coupled receptor and suppression of vascular barrier function.

To determine if CD69 induction by TNFα is also observed in lung-derived ECs, we treated human lung microvascular EC with various doses of TNFα and examined the expression of CD69, S1PR1, and VE-Cadherin. As shown in Figure S2, TNFα induced CD69 levels in a dose-dependent manner. Concomitantly, S1PR1 internalization into intracellular vesicles was also observed, and VE-cadherin staining suggested junctional disruption with the appearance of intercellular gaps.

### CD69 induction in lung ECs *in vivo*

**Figure 3:**
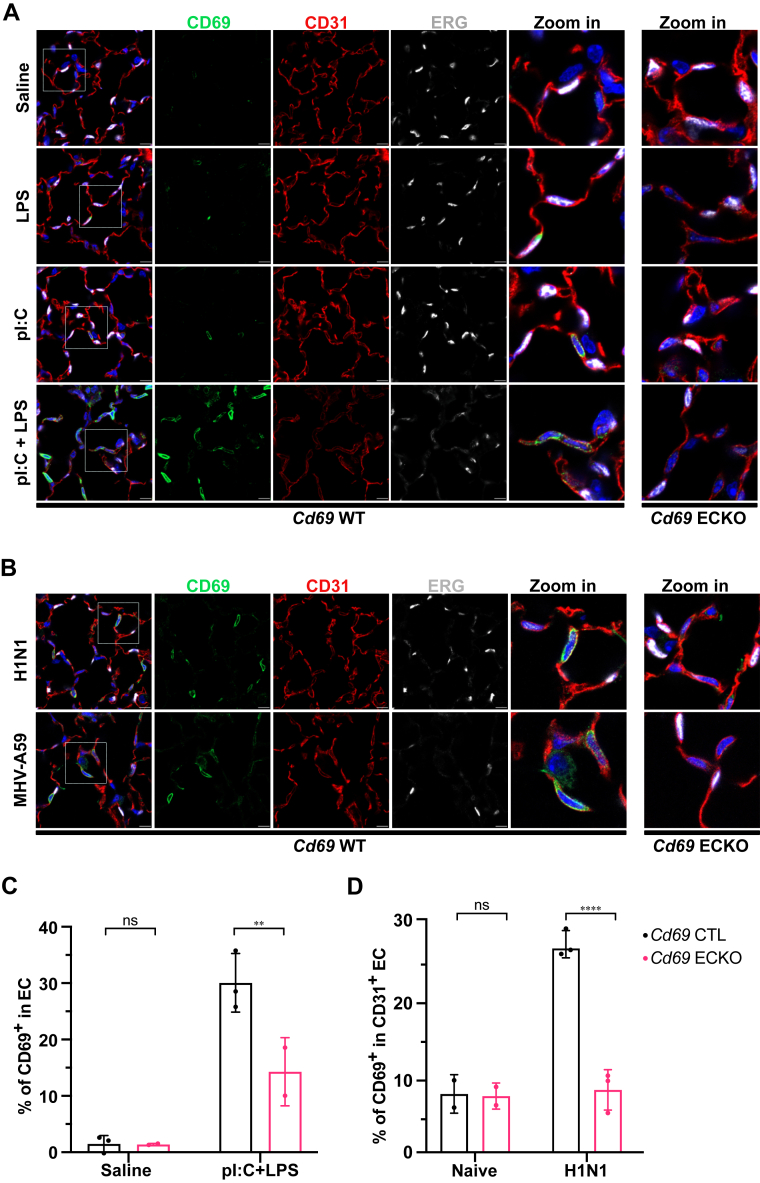
**CD69 expression is induced in lung ECs by systemic inflammation and respiratory viral infections**. *A*, *Cd69* WT and ECKO mice were treated with either LPS (10 mg/kg, 24 h), pI:C (8 mg/kg, four consecutive days) or both (LPS was given on the last 24 h of pI:C). Lungs were perfused, frozen in OCT, and sectioned (25 μm). Immunofluorescence images after staining with CD69 (*green*), CD31 (*red*), and ERG (*white*) antibodies were acquired by confocal microscopy. Representative images of three independent experiments are shown. The Zoom in for *Cd69* ECKO presented here is reused from Fig S3*B* for comparative purposes. *B*, Cd69 WT and *Cd69* ECKO mice were inoculated with either influenza A virus (H1N1) or murine hepatitis virus (MHV-A59), and lungs were harvested 7 days post infection. Lung sections were immunostained with CD69 (*green*), CD31 (*red*), and ERG (*white*) antibodies and imaged with confocal microscopy. Representative images from three independent infections are shown. The Zoom in for *Cd69* ECKO presented here is a reused from Fig S3*C* for comparative purposes. Quantification of CD69 expressing endothelial cells (CD31 and/or ERG) per field of view acquired at 63x were done for saline injected controls compared to pI:C + LPS animals (*C*) or naïve mice compared to H1N1 infected (*D*). Multiple fields (3–10) were acquired for each animal (N = 3) and results were analyzed by ordinary two-way ANOVA. (The scale bar represents 10 μm) ns = not significant; ∗∗ = *p* < 0.01; ∗∗∗∗ = *p* < 0.0001. EC, endothelial cell; ERG, ETS-related gene; LPS, lipopolysaccharide; OCT, optimal cutting temperature; pI:C, polyinosine/polycytosine.

To examine if CD69 is expressed in the lung endothelium *in vivo*, *Cd69* WT mice were treated with lipopolysaccharide (LPS, 10 mg/kg, *i*.*p*. for 12 h), or polyinosine/polycytosine (pI:C, 8 mg/kg/, *i*.*p*. daily for 4 days) or a combination of both (pI:C + LPS). CD69 expression was analyzed by immunofluorescence confocal microscopy of frozen sections of the lung and inguinal lymph node (positive control, Fig. S3*A*). Detection of EC markers CD31 and ERG were used together with CD69 antibody to determine CD69 expression in ECs (Fig. 3, *A* and *C*). Nontreated WT animals did not exhibit detectable CD69 in ECs (CD31^+^ ERG^+^). However, LPS or pI:C treatments induced CD69 expression in ECs. The combination treatment (pI:C + LPS) induced a higher frequency of CD69^bright^ and CD69^intermediate^ cells. The latter are also positive for CD31 and ERG suggesting that they are lung EC. Approximately 30% of CD31^+^ ERG^+^ cells are also CD69^intermediate^ in the acquired fields of the lungs. These cells however express lower levels of ERG. The strong induction of CD69 in *Cd69* WT inguinal lymph node after LPS treatment (Fig. S3*A*) combined with the lack of CD69 expression in *Cd69* gKO (*Cd69*^*f/f*^ *Rosa26-Cre*^*ERT2*^) support the validity of both our immunostaining conditions (CD69 antibody specificity), and the efficacy of the Cd69^f/f^ allele to generate the KO of the *Cd69* gene. To confirm their identity as EC, we developed mice that lack CD69 in ECs (*Cd69*^*f/f*^ *CDH5-Cre*^*ERT2*^, referred to as *Cd69* ECKO). The *Cd69* ECKO mice had marked reduction in the frequency of CD69^+^ CD31^+^ ERG^+^ cells when stimulated with LPS, or pI:C, or both (Fig. S3*B*), confirming that CD69 is induced by TLR3 and TLR4 agonists in lung ECs in mice.

We next tested if viral infections induce CD69 expression in the lung endothelium in mice. For this purpose, we used influenza A virus (IAV, H1N1 PR8 strain) (30) or murine hepatitis virus (MHV-A59 strain) which is in the coronavirus family (31, 32). We infected both *Cd69* WT and *Cd69* ECKO with a sublethal dose of IAV (H1N1 PR8 strain) or MHV-A59, both *via* an intranasal route. CD69, CD31 and ERG triple positive cells were detected in the lungs 7 days post infection (dpi) (Fig. 3*B*). H1N1 infection led to ∼ 25% of lung alveolar EC expressing CD69 in the loci of infection, which was almost reduced to background levels in the *Cd69* ECKO mice (Fig. 3, *B* and *D*, Fig. S3*C*). These data suggest that respiratory viral infections induce EC CD69 expression. Finally, immunofluorescence of the IAV nucleoprotein NP revealed the induced CD69 expression is found in proximity to the viral protein, suggesting that the viral infection is responsible for the CD69 induction (Fig. S3*D*)

To test if the EC specific deletion of *Cd69* had an impact on the initial phases of the IAV infection, we monitored the body weight of the mice (Fig. S4*A*) infected with a LD75 dose of IAV and observed no significant difference in the *Cd69* ECKO animal compared to controls. We then tested a higher dose of virus (10 times the LD75) and measured body weight of the mice and the O_2_ saturation (Fig. S4*B*) over the course of the infection. For both parameters, there was no significant difference between the *Cd69* WT and *Cd69* ECKO mice. H1N1 protein coding transcripts were not altered by *Cd69* gene deletion in the endothelium (Fig. S4, *D*–*F*). These data suggest that endothelial CD69 induction by the influenza viral infection did not impact viral replication. Similar results were observed after MHV-A59 infection with no significant difference between *Cd69* WT and ECKO mice in weight loss and oxygen saturation at day 6 post infection (Fig. S4, *G*–*H*).

### CD69 expression in mouse lung EC leads to vascular leak

**Figure 4:**
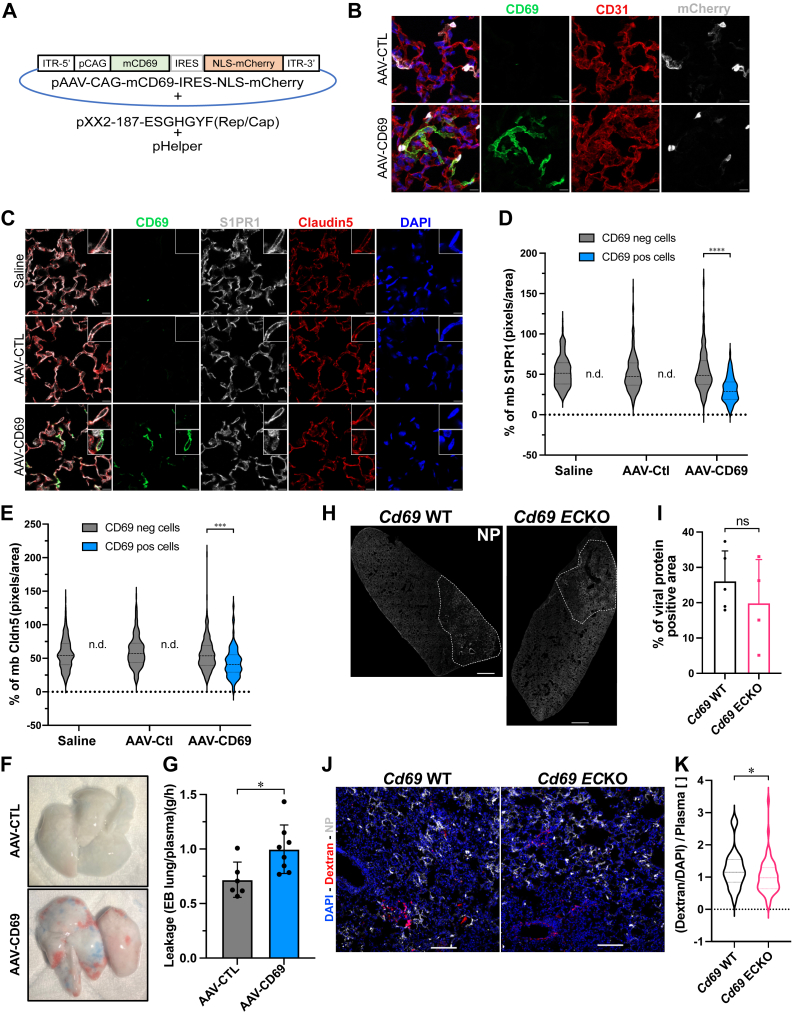
**CD69 expression in lung EC causes vascular leak**. *A*, map of the lung EC-targeted AAV vector containing *CD69* and NLS-mCherry reporter. C57BL/6 mice were injected intravenously (r.o.) with AAV-CTL or AAV-CD69, and lungs were harvested 4 weeks after infection. *B*, immunofluorescence staining with CD69 (*green*), CD31 (*red*), and mCherry (*white*) antibodies was done on lung sections and imaged by confocal microscopy (the scale bar represents 10 μm). Representative image from three independent infections is shown. *C*, AAV-infected lungs, and saline injected as control, were immunostained with S1PR1 (*white*), claudin5 (*red*), and CD69 (*green*) antibodies and imaged by confocal microscopy (the scale bar represents 10 μm). Representative image from three independent infections is shown. *D*–*E*, S1PR1 and claudin 5 immunoreactivity in EC plasma membrane in CD69^+^ and CD69^-^ cells were quantified by Fiji software with images from three separate infections. More than 30 fields and 300 cells were included, and 2-way ANOVA followed by Tukey multiple comparison test statistical analysis was performed. *F*, Evan’s blue extravasation was performed 4 weeks after administration of either AAV-CD69 or AAV-CTL and the quantification from two separate infection, six and eight mice per group, respectively, *G*, shows that AAV-CD69 infected lungs accumulated significantly more Evans Blue dye compared to the AAV-CTL. Unpaired *t* test statistical analysis was performed. *H*, confocal microscopy of H1N1-infected lungs from *Cd69* WT and *Cd69* ECKO mice. IAV nucleoprotein NP (*white*) staining is shown. *Dashed line* encircles the positive area of the viral protein immunoreactivity. Images are representative of five separate animals (the scale bar represents 1000 μm). *I*, quantification of the percent area of positive NP immunostaining on the total lung area. Statistical analysis was performed using a Mann-Whitney *t* test. *J*, *Cd69* WT and *Cd69* ECKO mice were injected with 70,000 MW dextran tetramethylrhodamine on day 7 post infection with IAV. Immunofluorescence for the IAV nucleoprotein (*white*) was performed combined with the detection of the dextran (*red*) and DAPI. Images shown are representative of five independent animals and more than 50 pictures taken across all lobes (the scale bar represents 100 μm). *K*, quantification of the dextran leakage was assessed throughout all lobes of the lungs of each animal, with 20 to 45 separate images taken randomly. The dextran pixels were normalized to the DAPI staining, and to the dextran concentration in the plasma. Statistical analysis was performed using a Mann–Whitney *t* test. ns = not significant; ∗ = *p* < 0.05; ∗∗∗ = *p* < 0.001; ∗∗∗∗ = *p* < 0.0001. AAV, adeno-associated virus; DAPI, 4',6-diamidino-2-phenylindole; EC, endothelial cell; IAV, influenza A virus; S1PR1, sphingosine 1-phosphate receptor-1.

We next tested the hypothesis that CD69 expression may induce lung vascular permeability. We overexpressed CD69 using the lung endothelial-specific adeno-associated virus (AAV) expression system that uses the AAV2-ESGHGYF capsid (33). The viral vector genome contains the mouse *Cd69* gene under the control of a constitutive promoter followed by an internal ribosome entry site (IRES) which permits the expression of a nuclear-targeted mCherry (NLS-mCherry) fluorescent protein (AAV-CD69) (Fig. 4*A*). As a negative control, we used the IRES-NLS-mCherry (AAV-CTL). Three weeks after systemic AAV transduction, both viruses induced nuclear mCherry expression in the lungs (Fig. 4*B*). Immunostaining for CD69 confirms the expression of CD69 is induced by the AAV-CD69 but not by the AAV-CTL. In combination with the CD31 costaining, we confirmed that CD69 is expressed in lung ECs. To test whether the overexpression of CD69 in ECs could lead to S1PR1 downregulation, we performed immunostaining for S1PR1 on sections of lungs receiving either AAVs and compared to saline injected counterparts. We observed reduced S1PR1 immunoreactivity in ECs that express CD69, compared to cells that do not (Fig. 4, *C* and *D*). The reduced expression of S1PR1 in ECs is not due to the AAV infection alone since in the AAV-CTL infected ECs, S1PR1 remained unaffected. We also assessed the effect of CD69 on the expression of endothelial tight junction protein claudin-5. Similar to S1PR1, claudin-5 expression was reduced in cells expressing CD69 (Fig. 4, *C*–*E*). However, immunostaining for EC adhesion protein VE-cadherin, or endothelial-specific nuclear marker ERG was unchanged in cells expressing CD69 (Fig. S5, *A* and *B*, respectively) when compared with CD69^-^ EC. These data suggest that induction of CD69 in lung alveolar EC reduced cell surface S1PR1 and claudin-5, which may impair tight junction function.

To determine if CD69 expression leads to vascular leak, we injected Evan’s blue dye (EBD) intravenously, perfused the circulatory system and measured retention of the EBD in the lung parenchyma. The lungs of mice receiving the AAV-CD69 accumulated significantly more EBD compared to the AAV-CTL (Fig. 4, *F* and *G*).

**Figure 5:**
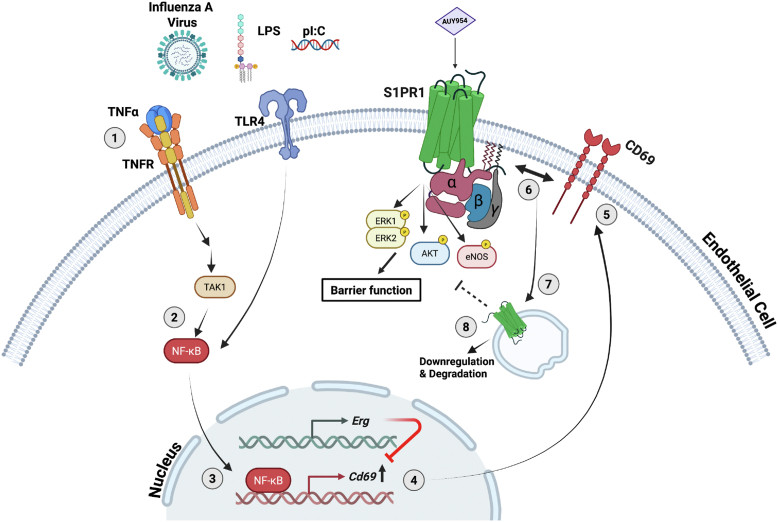
**Proposed model of the CD69-induced regulation of vascular permeability through S1PR1 downregulation**. S1PR1 activation by its specific agonist AUY954 induces a signaling cascade that involves the phosphorylation of ERK1/2, AKT, and eNOS. This activation of S1PR1 positively mediates the barrier function of endothelial cells. In this study, we showed that the activated TNFR and/or TLR4 cascades in ECs (by TNFα, LPS/pI:C, or viral infection) (1), leads to the induction of CD69 expression *via* NFkB and TAK1 (2, 3), and that the ERG downregulation facilitates this induction (4). Our data suggest the induced cell surface expression of CD69 (5) and its interaction with S1PR1 (6) leads to the downregulation of S1PR1 from the cell surface (7) and guides its degradation (8), which results in the decreased signaling of S1PR1 through the phosphorylation of ERK1/2, AKT, and eNOS, and its barrier function. ERG, ETS-related gene; LPS, lipopolysaccharide; pI:C, polyinosine/polycytosine; S1PR1, sphingosine 1-phosphate receptor-1; TLR4, toll-like receptor 4; TNFR, tumor necrosis factor receptor; TNFα, tumor necrosis factor α.

To determine if CD69 expression in lung EC influences vascular leak into the lung parenchyma, we infected *Cd69*^*f/f*^:*Cdh5-Cre*^*ERT2*^ (*Cd69* ECKO) and the *Cd69*^*f/f*^ (*Cd69* WT) mice with H1N1 virus and determined vascular leak into the lung parenchyma at day 7 post infection as described in the Experimental procedures. The extent of H1N1 infection of the lungs was similar as determined by H1N1 nucleoprotein (NP) immunostaining (Fig. 4, *H* and *I*). *Cd69* ECKO mice showed a significant decrease in lung leakage of fluorescent dextran (Fig. 4, *J* and *K*), suggesting that the lack of CD69 protein in the endothelium reduces vascular leak. Together, these results suggest that EC CD69 reduces S1PR1-dependent lung endothelial barrier integrity and increases vascular leak, as shown in the proposed model in Figure 5.

## Discussion

Our studies show that the lymphocyte activation molecule CD69 is induced by inflammatory cytokine signaling in EC. We also show that the canonical NFκB pathway, in which inflammatory receptors induce TAK-1/IKK kinase cascade involved in CD69 induction. In contrast to other cytokine inducible molecules such as VCAM-1, ICAM-1, or E-selectin, CD69 induction is restrained by the EC pioneer transcription factor ERG after TNF-α induction. It is of interest that other molecules that endow endothelial functionality and resilience (VEGFR2, VE-cadherin, claudin-5, and thrombomodulin) are maintained by ERG, whereas molecules associated with and/or drive endothelial injury (CXCL8, SERPINE1, VCAM1, and MMP3) are repressed by ERG, like CD69. Thus, CD69 could be considered as a unique marker of injured or dysfunctional endothelium. Further studies are needed to explore why CD69 induction is restrained in EC vis-à-vis immune cells, in which it is readily induced by cytokines expressed during immune activation.

Our data indicate that EC CD69 internalizes cell-surface S1PR1 and likely induces its proteasomal/lysosomal degradation. This results in abrogation of S1PR1 signals such as ERK and Akt phosphorylation. These pathways are needed for EC survival, proliferation after EC injury/death and eNOS activation, resulting in NO production. One of the key functions of the healthy EC is to maintain vascular barrier function, which is determined largely by the cell-cell adhesion molecule VE-cadherin and tight junctions that are formed by claudins and occludins. CD69 expressing ECs show defective vascular barrier function *in vitro*, suggesting that it may initiate vascular leak associated with acute inflammation. Thus, CD69 expressing endothelium is expected to be injured and dysfunctional.

Vascular EC dysfunction is important during respiratory viral infections. Viral infections of the airways induce mucosal injury that induces inflammatory cytokines such as TNF-α and interferons. Activation of cytokine receptors and TLRs during viral infection activates ECs to prepare the host to fight the infection. This involves adhesion molecule expression that helps recruits leukocytes needed for host defense, as well as transient loss of vascular barrier function leading to vascular leak that initiates inflammation of the tissue parenchyma. However, excessive activation of EC results in excessive vascular leak which is detrimental to the host. Our results show that CD69 is induced in the lung vascular endothelium after TLR3/4 activation, as well as after respiratory viral infections. Both mouse-adapted influenza and coronavirus infections induce CD69 in EC of the lung. Even though lack of CD69 in EC did not impair viral replication or acute viral infection symptoms (O_2_ saturation and weight loss), hypothetically because of the low percentage of lung ECs expressing CD69, AAV-induced expression of CD69 in lung ECs showed less S1PR1 and claudin-5 levels, suggesting junctional dysfunction and enhanced vascular leak. We did not observe significant alterations in VE-cadherin staining of the lung ECs *in vivo*. This may be related to the sensitivity of the assay which likely cannot distinguish between junctional versus endocytosed VE-cadherin. Nevertheless, EC-specific expression of CD69 leads to significant increase in lung vascular permeability. Therefore, our results suggest that cytokine activation results in CD69 expression that drives dysfunctional EC phenotypes. The acute and chronic consequences of CD69 function in EC need further investigation.

## Experimental procedures

### Reagents

Human recombinant TNFα was purchased from PeproTech, human recombinant Light from Biolegend, BAY11 to 7082, *Escherichia coli* LPS 0111:B4 (Sigma-Aldrich), Polyinosinic–polycytidylic acid sodium salt (poly(I:C)) from Sigma-Aldrich, AUY954 from Cellagen Technology, FBS from Corning, MG-132 from Enzo and 70,000 MW dextran, tetramethylrhodamine from Thermo Fisher Scientific. ERG ON-TARGETplus siRNA Reagents Human (siERG) and ON-TARGETplus Non-targeting Control Pool (siCTL) were ordered from Dharmacon. Antibodies for Flow Cytometry: CD69-PE (Clone FN50, Biolegend), S1PR1 monoclonal eFluor660 (SW4GYPP, eBioscience). Antibodies for western blot: anti-hCD69, anti-S1PR1 (H60, Santa Cruz Biotechnology), p-eNOS, total eNOS, p-Akt, total Akt, p-ERK1/2, total ERK1/2, ICAM1 (Cell Signaling Technology), anti-hVE-Cadherin (R&D), anti-HA, and anti-β-Actin (Sigma-Aldrich). Horseradish peroxidase (HRP)-conjugated anti-mouse, anti-rabbit and anti-rat IgG (Jackson ImmunoResearch). Antibodies for immunofluorescence: anti-S1PR1 (H60, Santa Cruz Biotechnology), S1PR1 monoclonal eFluor660 for cell surface (SW4GYPP, eBioscience), anti-mVE-cadherin (eBioBV13), anti-mClaudin 5 (Thermo Fisher Scientific), anti-mS1PR1, anti-mVE-cadherin, anti-mCD69 (R&D), anti-mCherry (Rockland Immunochemicals), anti-mPECAM-1 (MEC13.3; BD Pharmingen); anti-mERG (EPR3864; Abcam), IAV anti-nucleoprotein (GeneTex), and 4',6-diamidino-2-phenylindole (DAPI) (Invitrogen). The Alexa Fluor secondary antibodies used for immunofluorescence were purchased from Thermo Fisher Scientific: donkey anti-rabbit 488, 546, and 647, donkey anti-goat 488 and 546 and donkey anti-rat 550 and 647. DAPI was purchased from Invitrogen.

### Mice

Mice were housed in a temperature-controlled facility with a 12 h light/dark cycle, specific pathogen free, in individual ventilated cages and were provided food and water *ad libitum*. All animal experiments were approved by the Boston Children’s Hospital Institutional Animal Care and Use Committees. Global and EC-specific CD69 knockout mice (*Cd69*^*f/f*^ *Rosa26-Cre*^*ERT2*^; *Cd69* gKO and *Cd69*^*f/f*^ *Cdh5-Cre*^*ERT2*^; *Cd69* ECKO, respectively) were generated by using embryonic stem cells *Cd69*^*tm1a(KOMP)Wtsi*^ purchased from UC Davis KOMP Repository. The stem cells were treated with flippase to remove the lac Z reporter gene and to create a functional *Cd69*^*f/f*^ allele. Cells were then microinjected in C57BL/6 blastocyst-stage embryos then transferred in pseudopregnant surrogate mothers to create the *Cd69*^*f/f*^ mice. The *Cd69*^*f/f*^ mice were then crossed with *Rosa26-Cre*^*ERT2*^ or *Cdh5-Cre*^*ERT2*^ mice to generate global or endothelial specific inducible *Cd69* gKO and *Cd69* ECKO mice, respectively. Gene deletion by the CRE recombinase was achieved by gavage (p.o.) administration of tamoxifen (Sigma-Aldrich) (150 μg/g body weight per day) at 6 weeks of age for five consecutive days, and mice were allowed to recover for 2 weeks before being used for experiments. Littermates without the *Rosa26-Cre*^*ERT2*^ or *Cdh5-Cre*^*ERT2*^ gene were treated with tamoxifen the same way and used as controls. All genotyping was done by PCR using ear punch biopsies, and the primers used for genotyping before and after cre recombinase are listed in the Table S1. For Evan’s blue assay, C57BL/6J male mice were purchased at 7 weeks old from The Jackson Laboratory.

### Cell culture

HUVECs were grown in M199 (Corning), supplemented with 10% heat-inactivated FBS, EC growth factor from bovine brain extract, and 5 U/ml heparin (HUVEC medium), on human fibronectin–coated dishes, in a 37 °C incubator with 5% CO2 as previously described (T. Hla, T. Maciag, JBC. (1990)). Experiments were performed on HUVECs that were between passages 4 to 8. HEK293T cells were cultured in Dulbecco's modified Eagle's medium with L-glutamine, high glucose, and sodium pyruvate medium (Corning) supplemented with 10% FBS (293T medium) and in a 37 °C incubator with 5% CO2.

### RNA isolation and quantitative real-time PCR

At harvest time, cells were washed with 1X PBS directly in their tissue culture dish. Cell lysis was then initiated by adding TRI reagent (Zymo Research) and incubated with gentle agitation for 5 min at room temperature (RT). For mice lungs, whole tissues were flash frozen in liquid nitrogen and crushed with pilon and mortar. TRI reagent cell lysate were then collected by pipetting up and down multiple time and transferred to microtubes. Cell lysates were either stored frozen or directly processed for RNA extraction. Total RNA was isolated using Direct-zol RNA MicroPrep kit (Zymo Research) as recommended I by the manufacturer. Potential genomic DNA contamination was removed by column DNase 1 (30 U/μg total RNA, QIAGEN) digestion. Complementary DNA (cDNA) was generated from equal amount of total RNA using qScript XLT cDNA SuperMix (Quanta Bioscience). Expression of mRNA was quantitated using PerfeCTa SYBR Green FastMix Reaction Mixes (Quanta Bioscience) and a StepOnePlus or QuantStudio 6 Flex Real-Time PCR System (Applied Biosystems) with cDNA equivalent to 7.5 ng total RNA. Fold change level for each target gene was calculated using the ΔΔCt method. The reference gene used was *YWHAZ*. The statistical analysis was perform using two-way ANOVA followed by a *post hoc* Holm-Šídák’s multiple comparison test model on ΔΔCt values as they fit more accurately a normal distribution compared to fold changes which is affected by an exponential function. All primers used for quantitative PCR are presented in Table S2.

### Gene overexpression and KO in HUVEC

For overexpression of human CD69 and CLEC2A (control), the cDNA sequence of each gene were PCR-amplified from a plasmid template (CD69, pSport1-hCD69, and CLEC2A, Addgene #22851) and subcloned in the pCDH-puro vector. In the process, an HA-tag sequence was added in C terminus of both genes. Lentiviral particles were produced in HEK293T cells, seeded the day before transfection and grown to ∼70% confluency. One hour before transfection, medium was replaced by fresh 293T medium. For the transfection of each 10 cm dish, 15 μg of lentiviral plasmid (vector of interest), 15 μg pMDL/pRRE, 3 μg pVSV-G, and 6 μg pRSV-REV were diluted with water and mixed with 85.25 μl of 2 M CaCl_2_ solution for a final volume of 688 μl. The DNA/CaCl_2_ solution was slowly added to 688 μl of 2x HBS solution (274 mM NaCl, 1.5 mM Na_2_HPO_4_-7H_2_O, and 55 mM Hepes, pH 7.0). The solution mixture was incubated for 20 min at RT before being added to the cells drop-wise. The transfection medium was replaced by fresh 293T medium at 12 to 16 h post transfection. The lentiviral particle-containing supernatant was harvested 48 h after the medium exchange and filtered with a 0.45 μm filter (Corning). Lentivirus particles were then concentrated using PEG-it Virus Precipitation solution (System Biosciences). For the transduction of HUVEC, cells were seeded in HUVEC medium 24 h before being replaced by HUVEC medium containing the lentiviruses. The medium was renewed 1 day after transduction. The selection of the transduced cell population was initiated the following day by adding puromycin (2 μg/ml) and was maintained for further culture of the cells.

For gene knockouts in HUVEC, we used the CRISPR/Cas9 system as we previously published (34). Briefly, we cloned guide RNA (gRNA) sequences (see Table S3 for sequences) in the lentiCRISPRv2 vector (a gift from F. Zhang; Addgene plasmid #529619) (35) and packaged the corresponding lentiviruses as described above. Then, HUVEC cells were transduced at high multiplicity of infection and we selected the cells with puromycin. Our KO strategy was based on a combination of four gRNAs per gene. All four plasmids for one target gene were cotransfected in the same plate to produce a mixture of packaged lentiviruses encoding for all four gRNAs targeting one gene. The cotransfection was performed has described above, except that the amount of DNA allocated for the lentiviral plasmid (15ug) was evenly split between each four gRNA vectors for a given gene. HUVEC transduction was performed using the pooled lentiviruses to generate a nearly complete gene KO, as described above. The combination of gRNAs was selected in order to induce early translation termination due to the InDels (insertion/deletion) created by the gRNA/Cas9 cleavage. When allowed by the target gene genomic DNA sequence, we targeted the ATG start codon and/or an early splicing site that would render the translation of the full-length protein impossible. We also combined gRNAs with binding sites that spread across the gene body so that if an InDel was to create a frameshift, the alternative reading frame would present a “stop” codon before the next gRNA binding site. This strategy ensures that the series of InDels induced by the gRNA/Cas9 cleavage will prevent the translation of the full-length protein of the targeted gene. In the case of Hu-ERG-KO cells (Fig. 1, *I* and *J*), a single gRNA targeting the exon1 splicing donor site was used.

### Immunoblot analysis

Cells were washed with cold PBS and lysed in modified RIPA buffer (50 mM Tris, pH 7.4, 100 mM sodium chloride, 2 mM EDTA, 1% Triton X-100, 0.5% Fos-Choline, and 10 mM sodium azide) containing phosphatase inhibitors (1 mM sodium orthovanadate, 1mM sodium fluoride, and 5 mM β-glycerophosphate) and protease inhibitor cocktail (Sigma-Aldrich). After incubation on ice for 30 min and a freeze/thaw cycle, cell lysates were cleared of debris by centrifugation at 10,000 g (15 min at 4 °C). The protein concentrations of supernatants were determined by bicinchoninic acid assay (Pierce) and denatured for 30 min at RT in Laemmli’s sample buffer supplemented with 10% β-mercaptoethanol. An equal amount of protein was loaded and separated on an SDS polyacrylamide gel and transferred electrophoretically to a polyvinylidene difluoride membrane (Millipore). Blots were blocked in a solution of 4% skim milk in TBS-T or Intercept (TBS) Blocking Buffer (LI-COR) prior to incubation with primary antibody in fresh blocking buffer. Following primary antibody incubation, blots were washed with TBS-T and probed with the corresponding HRP-bound secondary antibodies before being revealed with Immobilon Western Chemiluminescent HRP Substrate and Bio-Rad Chemidoc Imager. Quantification was performed with Image Lab software (Bio-Rad).

### Flow cytometry analysis

HUVEC cells were detached with PBS containing EDTA/EGTA 2 mM each, at RT and stained on ice in FACS buffer (PBS/0.5% fatty acid-free bovine serum albumin [BSA]). The harvested cells were labeled with either PE anti-human CD69 (clone FN50, BioLegend) and/or eFluor 660 S1PR1 (clone SW4GYPP, eBioscience) on ice for 30 min. The samples were analyzed using a BD Calibur FACS system, and FlowJo software was used for data analysis.

### Measurement of endothelial barrier function *in vitro*

Endothelial barrier function was evaluated by measuring the resistance of a cell-covered electrode using an EC impedance system Zθ device (Applied BioPhysics) in accordance with the manufacturer’s instructions. Briefly, arrays were cleaned with 10 mM L-cysteine, washed with sterile water, coated with fibronectin for 30 min at 37 °C, and incubated with complete cell culture medium to run electrical stabilization. HUVECs were seeded on a 96-well electrode array (96W10idf) at a density of 2.5 x 10^4^ cell per well. The following day, dimethyl sulfoxide or AUY954 was diluted in M199/0.1% fatty acid-free BSA. Cells were stimulated by adding diluted ligand or vehicle to spent media (∼12–18 h old) to reach a final concentration of 100 nM. Resistance was monitored and expressed as fractional resistance, normalizing to the baseline at time 0. The average resistance of the last 20 measurements prior to stimulation was calculated for each well and used as baseline. AUC was calculated with Prism from the −30 min mark to +2 h post stimulation.

### H1N1 influenza A virus preparation and infection

Mouse-adapted H1N1 influenza A virus (PR/8/34) was obtained from Charles River (Catalog no. 10100374). For lethal dose (LD75), the viral stocks were calculated to contain 60,000 IU per mouse and diluted to a volume of 20 μl/mouse in PBS (30). Mice were treated intranasally under light anesthesia (ketamine/xylazine mix) on day 0 of the experiment and were monitored on a daily basis for signs of infection. The weights of the mice were recorded every day, and once the weight loss exceeded 20%–25%, the animal was euthanized. The maximum end point of the experiments was set at day 12, to capture the peak of inflammation. For dextran leakage, mice were injected *r*.*o*. with 0.25 mg of 70,000 MW dextran tetramethylrhodamine on day 7 of the infection. The dextran was left to circulate for 60 min, after which the mice were euthanized. Following perfusion with PBS, the lungs were inflated and processed for fluorescence confocal microscopy. The leakage was calculated as the amount of dextran (pixel) per field/DAPI pixels/field, on plasma concentration and time of circulation (Lung Dextran/DAPI)/(Plasma Dextran x time(h). For each animal (five per genotype), all lobes were imaged, and 25 to 40 images were taken throughout in regions where the nucleoprotein could be observed.

### MHV-A59 virus preparation and infection

MHV-A59 were purchased from BEI resources (NR-43000) and generated by infecting confluent monolayers of L2Percy cells. Forty-eight hours post inoculation, when >75% of the cells are present in syncytia, infected cells are be scraped into the media. The cell/supernatant mixture are frozen at – 80 °C to release virus from infected. Stocks were titrated using a tissue culture infectious dose 50 assay or plaque assay in L2Percy cells. 7e3 PFU inoculums were brought to a total volume of 40 μl in normal saline (mock normal saline for uninfected controls) and were pipetted dropwise into the nares of mice under light anesthesia (Ketamine 16 mg/Kg – Xylazine 3 mg/Kg). After the infection, body weight and vital signs including blood oxygen saturation were monitored.

### Oxygen pulse meter

Oxygen saturation was measured during the ongoing viral infection every other day. Precisely, mice were shaved around the neck area where the O_2_ sensor was applied. The measurements were recorded awake, using the MouseOx Plus (Starr Life Sciences Corp).

### Lung inflation and harvest for imaging

Mice were euthanized by ketamine overdose, and lungs were perfused through the heart with 10 ml 1x PBS under constant pressure. Paraformaldehyde (PFA) 4% in 1X PBS was then introduced *via* the exposed trachea to inflate the lungs under gravity pressure for 5 min. Briefly, the constant pressure inflation was achieved using 20 ml of PFA 4% solution in a 30 ml syringe with a one way stop valve to control the flow and a tube to connect the valve and the catheter used to cannulate the trachea. The syringe was raised to maintain 25 cm elevation between the body of the mouse and the surface of the liquid. The trachea was tied with a suture wire before stopping the PFA inflation pressure. The whole lung with the tied trachea was dissected out and further fixed by soaking in ice cold PFA 4% in PBS for 15 min. After the fixation, the inflated lungs were transferred to PBS. The following days, lungs were incubated with a sucrose gradient (10%-20%-30%, 24 h each) prior to embedding in optimal cutting temperature for cryostat sectioning.

### Immunofluorescence, histology, imaging, and quantification

HUVECs were washed with cold PBS and fixed with 2% PFA for 10 min at RT. Cells were permeabilized with PBS-0.5% Triton at RT for 30 min, then blocked with PBS containing 1% BSA, 1% donkey serum and 0.5% Triton X-100. Incubation with primary antibodies was performed overnight at 4 °C. For surface expression of S1PR1 on HUVECs, cells were kept on ice without detaching them, blocked with our FACS buffer and stained using the S1PR1 flow antibody (clone SW4GYPP, eBioscience) which recognizes an extracellular epitope on S1PR1. After washing, cells were fixed, permeabilized and further stained with an antibody targeting VE-cadherin and counterstained with DAPI. Lung cryosections (14 μm) were permeabilized with PBS - 0.5% Triton at RT for 30 min, then blocked with PBS containing 1% BSA, 1% donkey serum, and 0.5% Triton X-100. Incubation with primary antibodies was performed overnight at 4 °C. Sections were washed with PBS/0.5% Triton and incubated with secondary antibodies for 2 h at RT. Confocal images were taken using a Zeiss LSM 800 with Airyscan confocal microscope. Image processing and quantification was performed using Fiji software (National Institutes of Health). CD69 was observed in regions where the viral protein was also detected. Images were taken randomly in the area of viral infection, and the number of EC (CD31^+^ & ERG^+^) that were positive for CD69 was counted. In the case of pI:C + LPS, images were randomly and blindly taken across the lung tissue.

### Adeno-associated virus vectors for CD69

The viral vector genome we used contains the mouse *Cd69* gene under the control of a constitutive promoter followed by an IRES which permits the expression of a nuclear-targeted mCherry (NLS-mCherry) fluorescent protein (AAV-CD69). As a negative control, we built a vector that lacks the CD69 transgene but still contains the IRES-NLS-mCherry sequence (AAV-CTL). AAVs particles were produced by cotransfection of 293T cells plated the day before in 15-cm tissue culture dishes. Briefly, AAV genome plasmids (AAV-CD69 or AAV-CTL) were transfected along with the lung EC specific Rep/Cap plasmid (pXX2-ESGHGYF) and the helper (pAdDeltaF6, Addgene #112867) plasmid using PEI MAX (MW 40,000, Polysciences Inc) (33). 293T medium was replaced after overnight incubation with the transfection mix. AAVs were harvested 72 h after transfection (48 h after replacing medium) from both the cells and their medium. Briefly, cells were detached from the plates in their 48 h old medium using a cell scraper. Cells and supernatant were separated by centrifugation (5 min at 300g) in conical tubes. In our hands, the supernatant contained a larger fraction of the AAVs particles compared to cell lysates therefore both materials were used to purify viruses. AAVs particles were precipitated from supernatant by the addition of PEG8000 and NaCl, 5 and 2.9 g per 50 ml, respectively. After incubation at 4 ˚C overnight under gentle inversion, the enrichment was completed by centrifugation for 45 to 60 min at 4500g and 4 ˚C. AAV enriched pellets were resuspended in 1X PBS-MK buffer (1X PBS, 10 mM MgCl_2_, and 25 mM KCl). In parallel, cell pellets were pooled in one tube and resuspended in 1.5 ml per 15 cm dish of AAV lysis buffer (150 mM NaCl and 20 mM tris pH 8.0 and filter sterilized). After resuspension in lysis buffer, cell suspension was subjected to three cycles of freeze (dry ice/ethanol bath) and thaw (37 ˚C water bath), approximately 10 to 15 min per step. Contaminant RNA and DNA were removed with a nuclease digestion. Briefly, cell lysates were supplemented with MgCl_2_ (2 mM) and Pierce Universal Nuclease for cell lysis (100–200 units/ml) and digested for 15 min at 37 ˚C. The cell lysis process was finalized by a step of Dounce homogenization on ice and cleared of cell debris by centrifugation. AAVs from the cleared cell lysates were enriched using the same precipitation method as described above for the 293T medium containing released AAV particles. All pellets enriched in AAV particle were resuspended and pooled in 1X PBS-MK before loading on iodixanol gradient (OptiPrep Density Gradient Medium, STEMCELL Technologies) and ultracentrifugation. The AAV containing fractions were pooled and diluted in AAV concentration buffer (10 mM Tris, 100 mM sodium citrate, 0.001% Pluronic F-68 and pH 8.0) before concentration and cleanup on Amicon filter column (MWCO 100 kDa). The viral titer of purified and concentrated AAVs was determined by quantitative PCR quantification of viral genome copies DNAse I protected.

Each mouse received a dose of 4.00E + 11 copies of viral genome of the control and CD69 AAVs. We tested two routes of administration, intratracheal (i.t.) and intravenous (i.v.), and the latter shown a significantly higher efficiency of gene induction in lung ECs. The immunofluorescence and Evan’s blue assay were performed 4 weeks after the administration of the AAVs. No morbidity or health issues were noticed after the AAVs injections.

### Evan’s blue dye extravasation

Mice were administered Evan’s blue intravenously (r.o.) with 150 μl of a 5 mg/ml solution and left to circulate for 45 min. Mice were then euthanized by ketamine overdose, blood was drawn *via* the vena cava, lungs were perfused with 10 ml of 1x PBS and harvested. The wet weight of the lungs was taken, and then the lungs were dried in an oven at 65 °C for 72 h. The dry weight of the lungs was used to measure the amount of formamide added to the dried tissues to extract the Evan’s blue (15 μl/mg of tissue). The Evan’s blue concentration in the lungs and plasma were measured with a spectrophotometer at 620 nm. Leakage is expressed as the amount of Evan’s blue per gram of lungs, on plasma concentration and time of circulation [Lung EB concentration (mg/ml)/Lung weight (mg)]/[plasma EB concentration (mg/ml) x circulation time (h)].

### Statistical analysis

Statistical analysis was performed using GraphPad Prism software, version 10.3.1. Unpaired *t* test was used for direct comparison of two groups. One-way ANOVA followed by a Tukey test or two-way ANOVA followed by Holm-Šídák’s multiple comparison or Tukey’s test to compare all groups were used to determine significance between three or more test groups. All values reported are means ± SD. All animal experiments used randomization to treatment groups and blinded assessment.

## Data availability

The data supporting the findings of this study are available in the methods and supporting information of this article.

## Supporting information

This article contains supporting information.

## Conflict of interest

The authors declare that they have no conflicts of interest with the contents of this article.
