# Supplementary material for: Inflamed endothelial cells express S1PR1 inhibitor CD69 to induce vascular leak
Source: J Biol Chem. 2025 Jul 4;301(8):110455. doi: 10.1016/j.jbc.2025.110455 (PMC12336701; doi:10.1016/j.jbc.2025.110455)
Supplement: Table S1 [file mmc1.pdf]

Supporting information Table S1. Sequence of primers used for CD69$^{f/f}$ genotyping

Primer Name	Sequence	Genotype	Forward Primer	Reverse Primer	Amplicon size (bp)	TA NEB TAQ	Tm
TH78_CD69_CSD-Cd69-ttR	GGTCAATTTTACCAAATTCTGAGTGC	Wildtype	CSD-Cd69-F	CSD-Cd69-ttR			
TH80_CD69_CSD-Cd69-F	ATTAATATATGAAAGTTTGCTGCACTGG	PostFlp & Cre	CSD-Cd69-F	CSD-Cd69-R	491	47˚C	52˚C
TH89_CD69_upstream-FRT-loxP_Fwd	AAGGCGCATAACGATACCAC						54˚C
TH90_CD69_loxP-downstream_Rev	GCAACTACTTCACCGCCCTT	PostFlp	2856 bp	PostFlp & Cre	TH89-90: 307 bp	49˚C	57˚C
TH91_CD69_Intron1_Rev	TGGAAAACGTCTTGTTGGCT			PostFlp	TH89-91: 443 bp	48˚C	53˚C
