# Supplementary material for: Inflamed endothelial cells express S1PR1 inhibitor CD69 to induce vascular leak
Source: J Biol Chem. 2025 Jul 4;301(8):110455. doi: 10.1016/j.jbc.2025.110455 (PMC12336701; doi:10.1016/j.jbc.2025.110455)
Supplement: Table S2 [file mmc2.pdf]

Supporting information Table S2. Sequence of primers used for qPCR

Target Gene	Primer Name	Sequence 5'→3'	Species	Direction	Source
YWHAZ	ML164_hYWHAZ_qPCR-Fwd	ACTTTTGGTACATTGTGGCTTCAA	Human	Forward	PMID:23251572
YWHAZ	ML165_hYWHAZ_qPCR-Rev	CCGCCAGGACAAACCAGTAT	Human	Reverse	PMID:23251572
CD69	ML284_hCD69_qPCR-Fwd	TCTACTGTGAAGAGGAGCTGGAC	Human	Forward	NIH Primer Blast
CD69	ML285_hCD69_qPCR-Rev	TTTCAGAATCAATGACAGCAAGA	Human	Reverse	NIH Primer Blast
VCAM1	ML290_VCAM-1_qPCR-Fwd	TTAAGGGGGAGACTATTCTGGAG	Human	Forward	NIH Primer Blast
VCAM1	ML291_VCAM-1_qPCR-Rev	TTCCAGTATCTTCAATGGTAGGG	Human	Reverse	NIH Primer Blast
ERG	ML504_hERG_qPCR_Fwd	GATCGCATTATGGCCAGCAC	Human	Forward	NIH Primer Blast
ERG	ML505_hERG_qPCR_Rev	CGTTCCGTAGGCACACTCAA	Human	Reverse	NIH Primer Blast
HPRT1	ML168_mHPRT_qPCR-FWD	CCTAAGATGAGCGCAAGTTGAA	Mosue	Forward	NIH Primer Blast
HPRT1	ML169_mHPRT_qPCR-Rev	CCACAGGACTAGAACACCTGCTAA	Mosue	Reverse	NIH Primer Blast
Polymerase gene	ML723_IAV-PA_qPCR_Fwd	CGGTCCAAATTCCTGCTGA	IAV-PR8_H1N1	Forward	NIH Primer Blast
Polymerase gene	ML724_IAV-PA_qPCR_Rev	CATTGGGTTCCTTCCATCCA	IAV-PR8_H1N1	Reverse	NIH Primer Blast
Nucleoprotein gene	ML725_IAV-NP_qPCR_Fwd	CAGCCTAATCAGACCAAATG	IAV-PR8_H1N1	Forward	NIH Primer Blast
Nucleoprotein gene	ML726_IAV-NP_qPCR_Rev	TACCTGCTTCTCAGTTCAAG	IAV-PR8_H1N1	Reverse	NIH Primer Blast
Hemagglutinin gene	ML727_IAV-HA_qPCR_Fwd	GAGGAGCTGAGGGAGCAAT	IAV-PR8_H1N1	Forward	NIH Primer Blast
Hemagglutinin gene	ML728_IAV-HA_qPCR_Rev	GCCGTTACTCCGTTTGTGTT	IAV-PR8_H1N1	Reverse	NIH Primer Blast
