# Supplementary material for: Inflamed endothelial cells express S1PR1 inhibitor CD69 to induce vascular leak
Source: J Biol Chem. 2025 Jul 4;301(8):110455. doi: 10.1016/j.jbc.2025.110455 (PMC12336701; doi:10.1016/j.jbc.2025.110455)
Supplement: Table S3 [file mmc3.pdf]

Supporting information Table S3. Sequence of gRNA primers

Gene	gRNA	Sequence 5'→3'
MAP3K7 (TAK1)	TAK1_Ex1_gRNAa	gatgatcgaagccccttccc
	TAK1_Ex3_gRNAb	aatattaggatggttcacac
	TAK1_Ex5_gRNAc	gtaaacaccaactcattgcg
	TAK1_Ex5_gRNAd	acccaaagcgctaattcaca
MAP3K14 (NIK)	NIK_Ex2_gRNAa	gagctccgtctacaagcttg
	NIK_Ex5_gRNAb	cgtggttcagacattgcaag
	NIK_Ex5_gRNAc	gggctttggactgtctacac
	NIK_Ex5_gRNAd	cccaaaactgaggacaacga
GFP control	GFP_gRNA	ggcgagggcgatgccaccta
