# Supplementary material for: Inflamed endothelial cells express S1PR1 inhibitor CD69 to induce vascular leak
Source: J Biol Chem. 2025 Jul 4;301(8):110455. doi: 10.1016/j.jbc.2025.110455 (PMC12336701; doi:10.1016/j.jbc.2025.110455)
Supplement: Figure S1 [file mmc4.pdf]

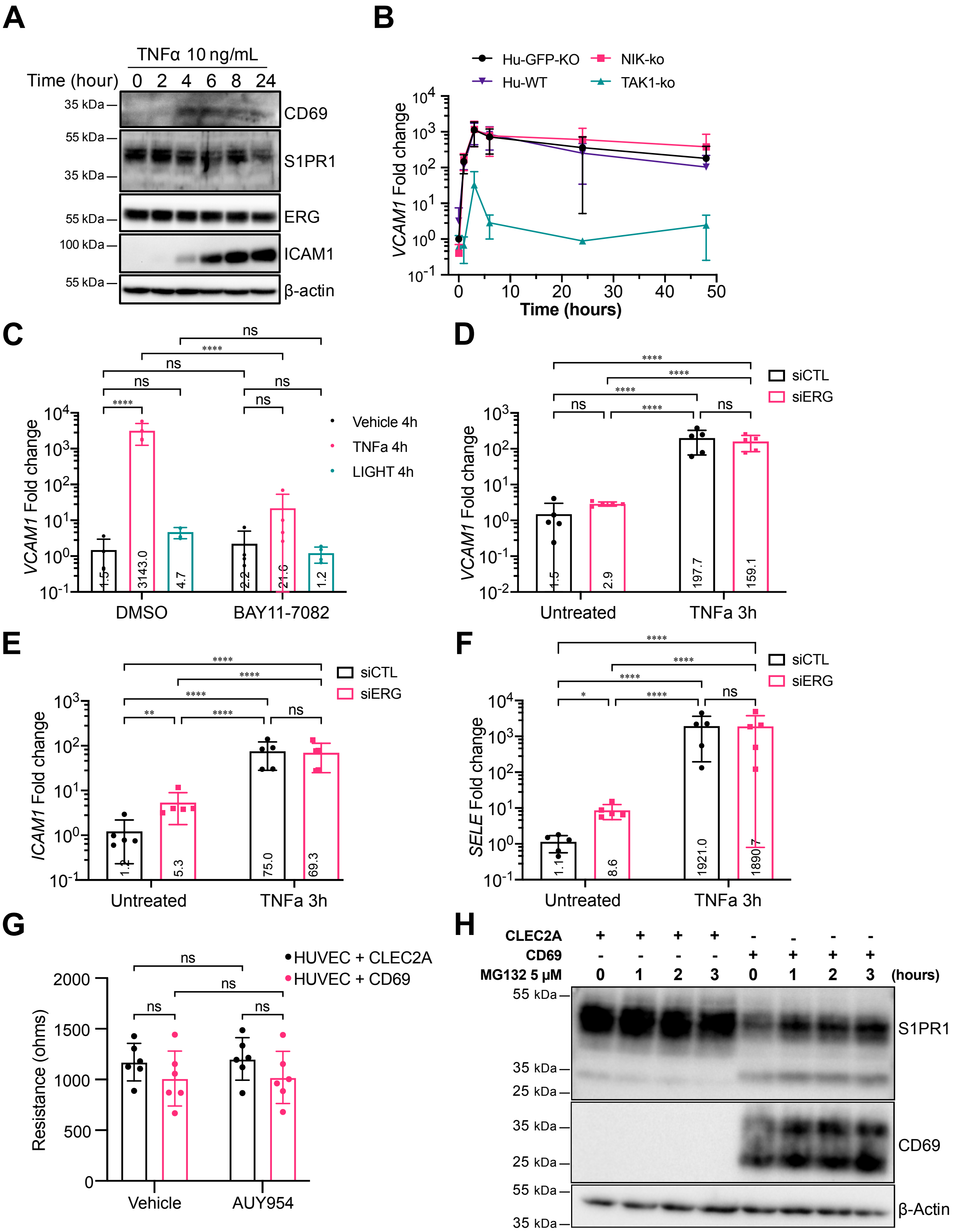

A
TNFα 10 ng/mL
Time (hour) 0 2 4 6 8 24
CD69
S1PR1
ERG
ICAM1
β-actin
B
Hu-GFP-KO
NIK-ko
Hu-WT
TAK1-ko
VCAM1 Fold change
Time (hours)
C
Vehicle 4h
TNFa 4h
LIGHT 4h
VCAM1 Fold change
DMSO
BAY11-7082
D
siCTL
siERG
VCAM1 Fold change
Untreated
TNFa 3h
E
ICAM1 Fold change
F
SELE Fold change
G
HUVEC + CLEC2A
HUVEC + CD69
Resistance (ohms)
Vehicle
AUY954
H
CLEC2A
CD69
MG132 5 μM
(hours)
S1PR1
CD69
β-Actin

Supporting information Figure S1. CD69 expression in HMVEC, VCAM1 expression, regulation of EC activated genes by ERG, and rescue of S1PR1 expression by MG132 in ECs *in vitro.*

(**A**) Immunoblot analysis of TNFα treated HMVEC-L at 10 ng/mL for the defined times for CD69, S1PR1, ERG, ICAM1 and β-Actin expression. (**B**) RT-qPCR analysis of *VCAM1* mRNA expression in HUVEC CRISPR/Cas9 knockout for *NIK* (NIK-ko) and *TAK1* (TAK1-ko). HUVEC expressing a gRNA targeting GFP (GFP-ko) and parental HUVEC (no transduction) were use as controls. Cells were treated with 10 ng/mL of TNFα for different times prior to total RNA harvest. CD69 expression is presented as change ($2^{-\Delta\Delta Ct}$) of control untreated cells and analyzed with two-way ANOVA followed by Holm-Šídák's multiple comparison test. (**C**) RT-qPCR analysis of *VCAM1* mRNA expression in HUVEC pre-treated for 1 hr with BAY11-7082 or DMSO (diluent) followed by treatment with vehicle (black), TNFα (pink) or LIGHT (green) for 4 hrs. Gene expression is presented as fold change ($2^{-\Delta\Delta Ct}$) of control untreated cells. Statistical analysis was performed on ΔΔCt values using a two-way ANOVA followed by a post-hoc Holm-Sidak test for multiple comparisons from four independent experiments. RT-qPCR analysis of *VCAM1* (**D**), *ICAM1* (**E**) and *SELE* (**F**) mRNA expression in HUVEC transfected with siRNA control (siCTL) or siRNA targeting ERG (siERG). After 48 hrs post-transfection, cells were treated 3 hrs with TNFα or vehicle (Untreated) prior to total RNA harvest. Gene expression is presented as fold change ($2^{-\Delta\Delta Ct}$) of control untreated cells from five independent experiments and analyzed with two-way ANOVA followed by Holm-Šídák's multiple comparison test. (**G**) HUVECs overexpressing CD69 or CLEC2A were analyzed for barrier function by real-time measurement of TEER. The resistance of each cell population for

both conditions (vehicle and AUY954) at the start of the TEER is shown. Statistical analysis of 6 independent experiments was performed using a two-way ANOVA followed by a post-hoc Holm-Šídák test for multiple comparisons. (**H**) Immunoblot analysis of S1PR1 in HUVECs overexpressing CD69 or CLEC2A treated with MG132 (5μM) for 0 to 3 hrs. Representative immunoblot of 2 independent experiments is shown. ns = not significant; ∗= p< 0.05; ∗∗= p< 0.01; ∗∗∗∗= p< 0.0001
