# Supplementary material for: Inflamed endothelial cells express S1PR1 inhibitor CD69 to induce vascular leak
Source: J Biol Chem. 2025 Jul 4;301(8):110455. doi: 10.1016/j.jbc.2025.110455 (PMC12336701; doi:10.1016/j.jbc.2025.110455)
Supplement: Figure S2 [file mmc5.pdf]

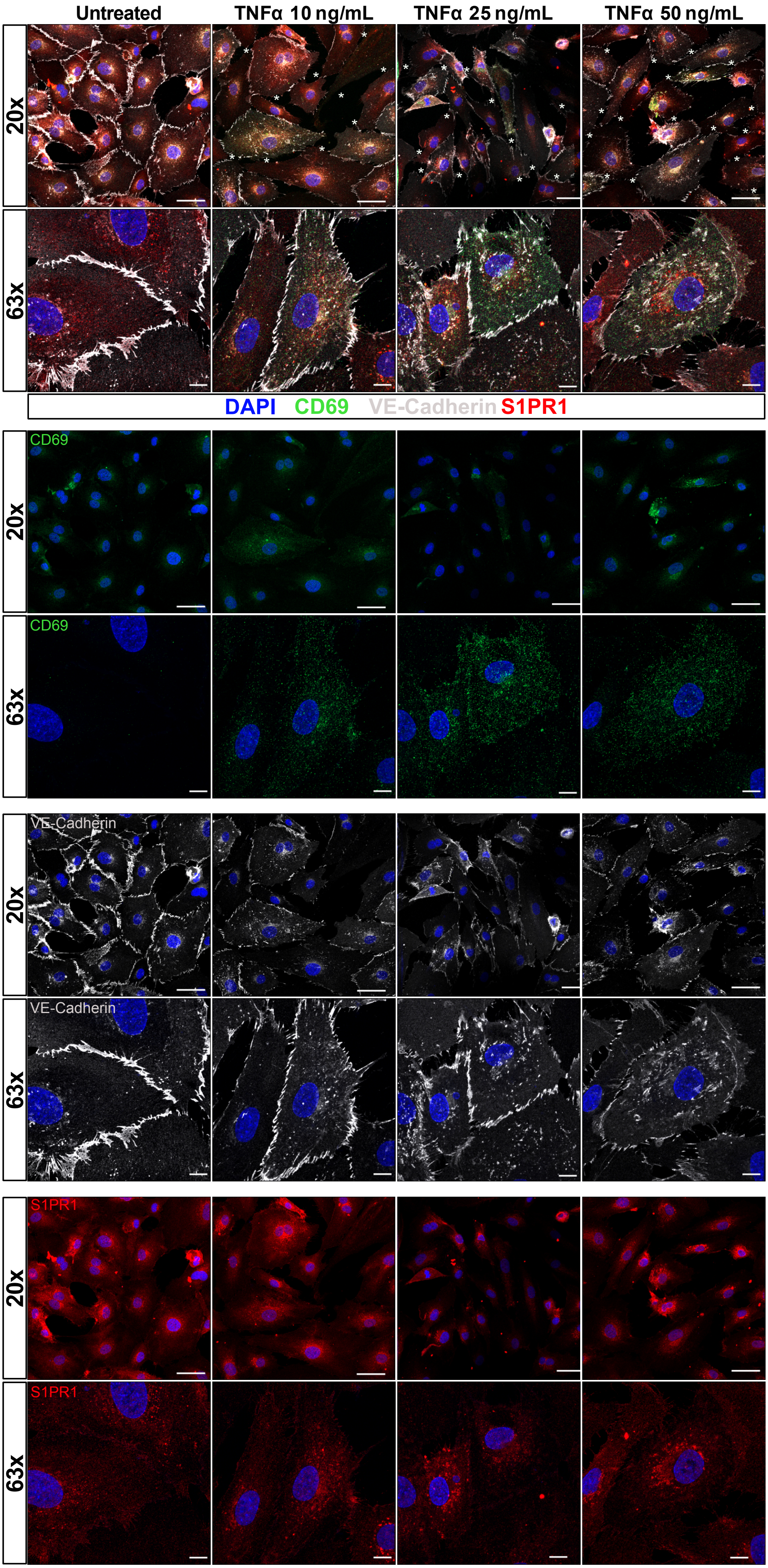
Untreated
TNFα 10 ng/mL
TNFα 25 ng/mL
TNFα 50 ng/mL
20x
63x
DAPI CD69 VE-Cadherin S1PR1
CD69
VE-Cadherin
S1PR1

Supporting information Figure S2. TNFα induction of CD69 in HMVEC-L.

Confocal microscopy of HMVEC-L cells treated with an increasing concentration of TNFα, for 24 hrs. Immunostaining of CD69 (green), S1PR1 (red), VE-Cadherin (white) and DAPI (blue) were performed. White asterisks mark intercellular gaps in the VE-Cadherin staining. (Scale bar = 50μm (20x) and 10μm (63x)). Representative images of 5 separate areas are shown.
