# Supplementary material for: Inflamed endothelial cells express S1PR1 inhibitor CD69 to induce vascular leak
Source: J Biol Chem. 2025 Jul 4;301(8):110455. doi: 10.1016/j.jbc.2025.110455 (PMC12336701; doi:10.1016/j.jbc.2025.110455)
Supplement: Figure S3 [file mmc6.pdf]

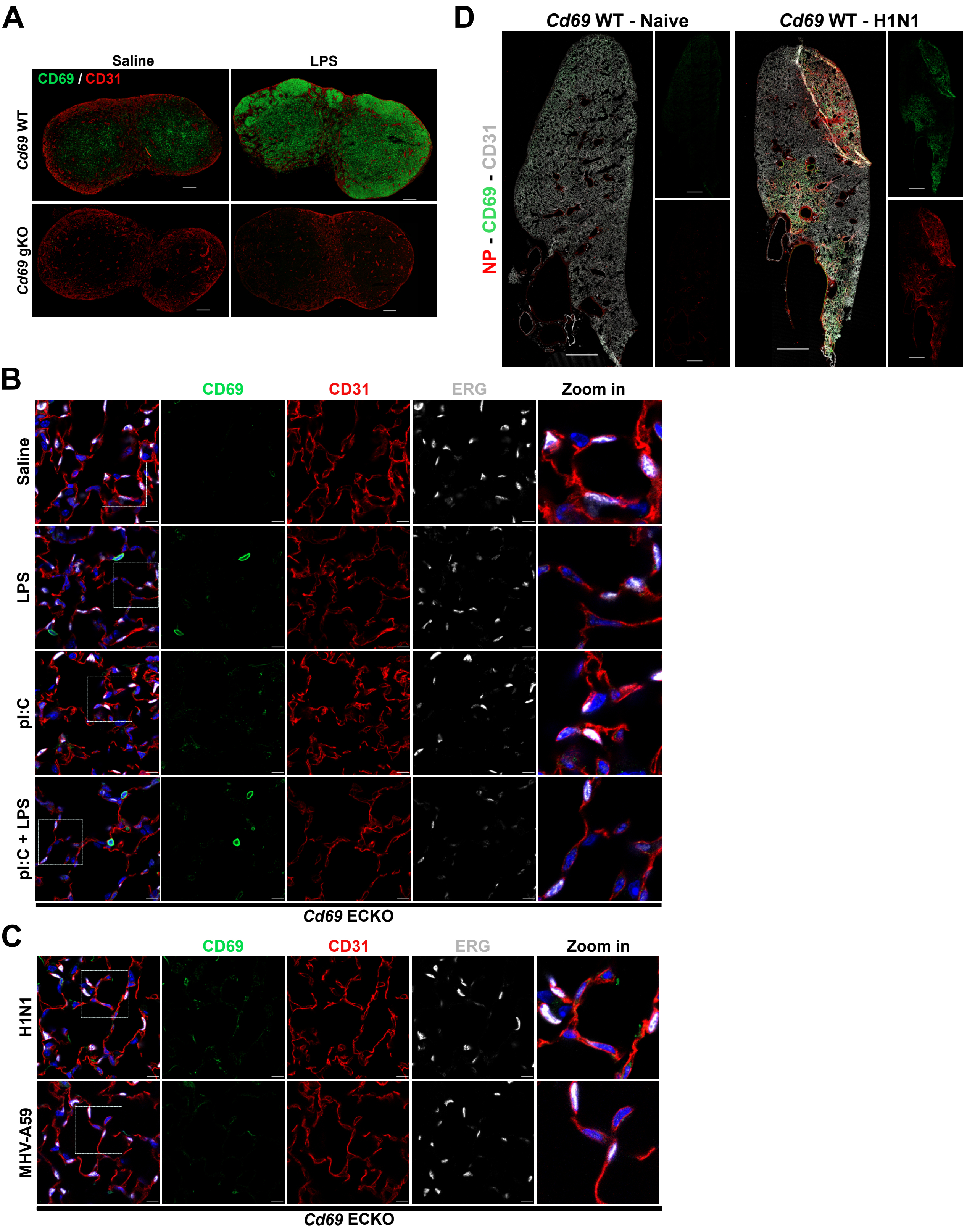

A
Saline
LPS
CD69 / CD31
Cd69 WT
Cd69 gKO
D
Cd69 WT - Naive
Cd69 WT - H1N1
NP - CD69 - CD31
B
CD69
CD31
ERG
Zoom in
Saline
LPS
pI:C
pI:C + LPS
Cd69 ECKO
C
CD69
CD31
ERG
Zoom in
H1N1
MHV-A59
Cd69 ECKO

Supporting information Figure S3. EC CD69 induction is not detectable in *Cd69* ECKO mice.

(**A**) Confocal microscopy of lymph nodes from *Cd69* gKO or *Cd69* WT mice treated with LPS 10mg/kg for 16 hrs. CD69 (green) and CD31 (red) staining are shown and images are representative of 3 separate experiments. (Scale = 200µm) (**B**) *Cd69* ECKO mice were treated with either LPS (10mg/kg, 24h), pI:C (8mg/kg, 4 consecutive days) or both (LPS was given on the last 24 hrs of pI:C). Saline was administered to the mice with the same regiment than pI:C and LPS and served as control. Lungs from each mouse group were perfused, frozen in OCT and sectioned (25 µm). Immunofluorescence staining with CD69 (green), CD31 (red) and ERG (white) antibodies was acquired by confocal microscopy. Representative images from 3 independent experiments are shown. Scale bar = 10µm) (**C**) *CD69* ECKO mice were inoculated with either Influenza A virus (H1N1) or Mouse Hepatitis Virus (MHV-A59), and lungs were harvested 7 days post-infection. Lung sections were immunostained with CD69 (green), CD31 (red) and ERG (white) antibodies. The immunofluorescence acquired by confocal microscopy shown no CD69 expression induced in endothelial cells (CD31+) following a viral infection in the ECKO mice. Representative images from 3 independent infections are shown. Scale bar = 10µm) (**D**) Confocal microscopy of naive and H1N1-infected lungs from *Cd69* WT mice 7 days post infection. CD69 (green), CD31 (white) and IAV nucleoprotein NP (red) staining are shown. Images are representative of 3 separate experiments. (Scale = 1000µm)
