# Supplementary material for: Inflamed endothelial cells express S1PR1 inhibitor CD69 to induce vascular leak
Source: J Biol Chem. 2025 Jul 4;301(8):110455. doi: 10.1016/j.jbc.2025.110455 (PMC12336701; doi:10.1016/j.jbc.2025.110455)
Supplement: Figure S4 [file mmc7.pdf]

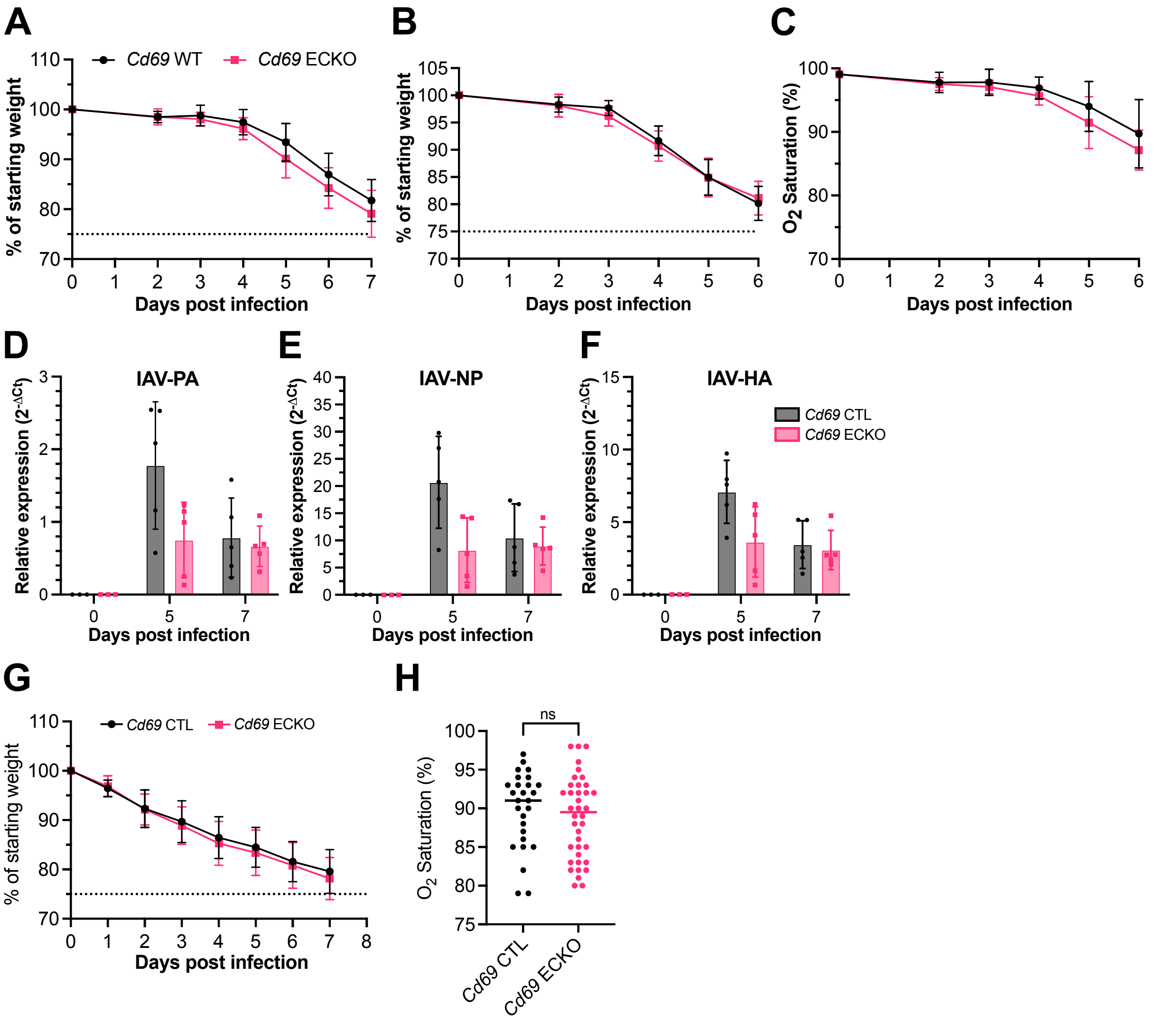

Supporting information Figure S4. EC CD69 induction did not impact viral infection parameters.

(**A**) Body weight of mice infected with a LD75 dose of IAV taken from two separate infections, with a total of 21 (WT) and 12 (ECKO) mice. (**B**) A higher dose of virus (10 times the LD75) was used, and the body weight of the mice and O_2 saturation percent (**C**) are shown for 5 (WT) and 6 (ECKO) mice. (**D-F**) qRT-PCR of mRNAs for H1N1 viral proteins (PA - Polymerase acidic, NP - nucleoprotein, HA - Hemagglutinin) present in the lungs of *Cd69* WT and ECKO at day 0, 5 and 7 post infection with LD75 dose. Data are from 3-5 mice per genotype, per time point and analyzed by two-way ANOVA followed by Tukey's multiple comparison test. (**G**) Weight loss and O_2 saturation (**H**) following MHV-A59 infection did not show a significant difference between *Cd69* WT and ECKO mice at day 6 post infection. Data shown are from three independent infections (27 mice for WT and 38 for ECKO), and O_2 at day 6 post infection was analyzed by unpaired t test. ns = not significant
