# Supplementary material for: Inflamed endothelial cells express S1PR1 inhibitor CD69 to induce vascular leak
Source: J Biol Chem. 2025 Jul 4;301(8):110455. doi: 10.1016/j.jbc.2025.110455 (PMC12336701; doi:10.1016/j.jbc.2025.110455)
Supplement: Figure S5 [file mmc8.pdf]

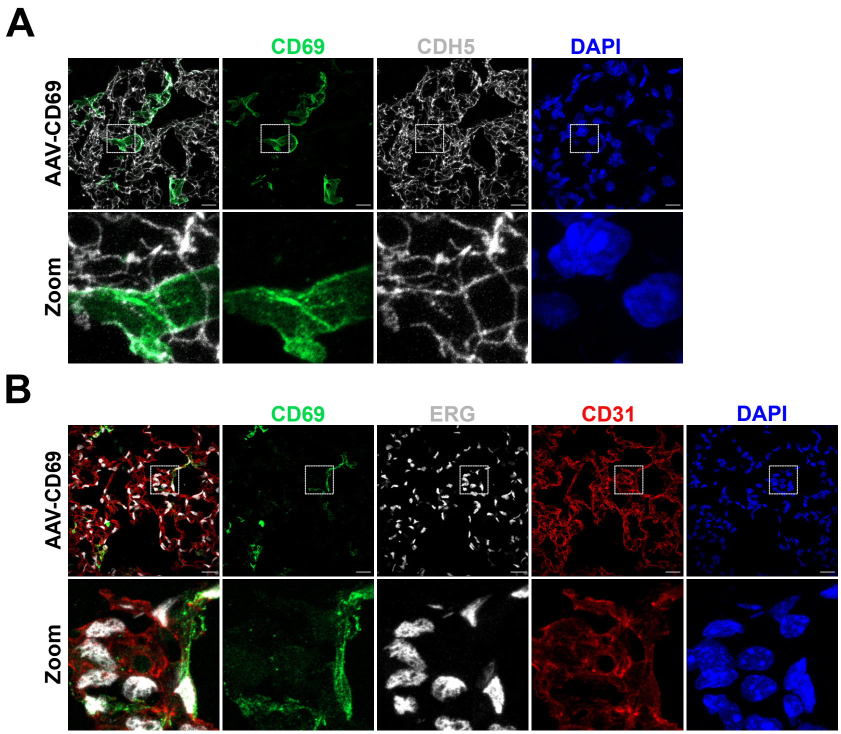

Supporting information Figure S5. EC specific adhesion and nuclear protein were unchanged by CD69 induction.

AV-CD69 infected lungs were immunostained with CDH5 (white) and CD69 (green) antibodies (**A**) or ERG (white), CD31 (red) and CD69 (green) (Scale bar = 10 μm) (**B**) and imaged by confocal microscopy. (Scale bar = 20 μm) Representative images from three independent infections are shown.
